# Rapid and long-term effects of water deficit on gas exchange and hydraulic conductance of silver birch trees grown under varying atmospheric humidity

**DOI:** 10.1186/1471-2229-14-72

**Published:** 2014-03-24

**Authors:** Arne Sellin, Aigar Niglas, Eele Õunapuu-Pikas, Priit Kupper

**Affiliations:** 1 Institute of Ecology and Earth Sciences, University of Tartu, Lai 40, Tartu 51005, Estonia

**Keywords:** *Betula pendula*, Branch water potential, Climate change, Hydraulic conductance, Leaf water potential, Net photosynthesis, Silver birch, Stomatal conductance, Water-use efficiency

## Abstract

**Background:**

Effects of water deficit on plant water status, gas exchange and hydraulic conductance were investigated in *Betula pendula* under artificially manipulated air humidity in Eastern Estonia. The study was aimed to broaden an understanding of the ability of trees to acclimate with the increasing atmospheric humidity predicted for northern Europe. Rapidly-induced water deficit was imposed by dehydrating cut branches in open-air conditions; long-term water deficit was generated by seasonal drought.

**Results:**

The rapid water deficit quantified by leaf (Ψ_L_) and branch water potentials (Ψ_B_) had a significant (*P* < 0.001) effect on gas exchange parameters, while inclusion of Ψ_B_ in models resulted in a considerably better fit than those including Ψ_L_, which supports the idea that stomatal openness is regulated to prevent stem rather than leaf xylem dysfunction. Under moderate water deficit (Ψ_L_≥-1.55 MPa), leaf conductance to water vapour (*g*_L_), transpiration rate and leaf hydraulic conductance (*K*_L_) were higher (*P* < 0.05) and leaf temperature lower in trees grown in elevated air humidity (**H** treatment) than in control trees (**C** treatment). Under severe water deficit (Ψ_L_<-1.55 MPa), the treatments showed no difference. The humidification manipulation influenced most of the studied characteristics, while the effect was to a great extent realized through changes in soil water availability, i.e. due to higher soil water potential in **H** treatment. Two functional characteristics (*g*_L_, *K*_L_) exhibited higher (*P* < 0.05) sensitivity to water deficit in trees grown under increased air humidity.

**Conclusions:**

The experiment supported the hypothesis that physiological traits in trees acclimated to higher air humidity exhibit higher sensitivity to rapid water deficit with respect to two characteristics - leaf conductance to water vapour and leaf hydraulic conductance. Disproportionate changes in sensitivity of stomatal versus leaf hydraulic conductance to water deficit will impose greater risk of desiccation-induced hydraulic dysfunction on the plants, grown under high atmospheric humidity, in case of sudden weather fluctuations, and might represent a potential threat in hemiboreal forest ecosystems. There is no trade-off between plant hydraulic capacity and photosynthetic water-use efficiency on short time scale.

## Background

Global warming is accompanied by changes in atmospheric water vapour content and precipitation rate, although there will be pronounced regional differences in their magnitude and direction [1]. Over a period from 1900 to 2005 precipitation has significantly increased in northern Europe and continuation of this trend with larger increase in the frequency than in the magnitude of precipitation is predicted from climatic models. Climate change scenarios predict by the end of the century increases in air temperature by 3.5–5ºC and precipitation by 5–30% in boreal and northern temperate regions of Europe [2,3]. Increase in atmospheric relative humidity (RH), the inevitable result of more frequent rainfall events, will reduce water loss through transpiration [4,5], and affect both the delivery of nutrients to root absorbing surface and nutrient uptake by trees due to diminished water fluxes through the vegetation [6,7].

On the other hand, climate extremes including heat waves and droughts across Europe are projected to become more frequent and enduring over the 21st century [1,8]. Because trees have adapted to local average climatic conditions, extreme events have consequences on forest health and productivity across site conditions [9,10]. Plants growing in humid air have less effective stomatal control over transpirational water loss [4,11,12] and demonstrate higher vulnerability to xylem cavitation, i.e. have narrow hydraulic safety margin [13,14]. In addition, Okamoto et al. [15] demonstrated that high air humidity induces abscisic acid (ABA) 8′-hydroxylase in stomata and vasculature, followed by the reduction of ABA levels - a plant hormone, which promotes stomatal closure under water deficit [16].

Water deficit decreases stomatal conductance before leaf water potential (Ψ_L_) falls below critical values, to avoid adverse consequences on leaf tissues (dehydration of protoplasm) and water transport system (hydraulic dysfunction through runaway xylem cavitation). However, the mechanisms by which stomata respond to and control Ψ_L_ are still unclear [14,17]. The classical view suggests that a primary signal of water shortage is ABA, produced by roots situated in dry soil and transported to shoots [18]. As a result, a considerable time lag is expected in the response of stomata to changing soil water status. Soil drying concentrates ABA in both the xylem sap and leaves [19-21]. This is followed by water efflux from guard cells and stomatal closure [22]. Stricter stomatal control leads to increasing short-term (intrinsic water-use efficiency [23]) and long-term water-use efficiency (carbon isotope discrimination [24]).

In *Arabidopsis*, shoot vascular tissues appear to be a major site of ABA biosynthesis and suggest tissue-autonomous ABA synthesis in addition to its long-distance root-to-shoot movement [16,25]. Bauer et al. [26] report that guard cells possess the entire ABA biosynthesis pathway and that cell-autonomous synthesis is sufficient for stomatal closure. Thus, effects of fast changes in leaf water status do not involve chemical signals from roots, but rather are predominantly hydraulic [22,27,28]. Guard cells respond to changes in Ψ_L_ either directly or via a signal generated close by [29]. Stomatal closure, in turn, will increase stomatal limitation to photosynthesis. At severe water deficit, efficiency of photosystem II will decrease as well [12,30,31] further impelling decline of CO_2_ assimilation.

The structure and function of the water transport system govern the productivity and survival of land plants because the vascular architecture places a physical limit on plant functioning [29,32]. Therefore, the water pathway from the soil-root interface to the sites of evaporation in leaves is critical to maintain leaf water status and hold stomata open, keeping a positive carbon budget. Water deficit will induce cavitation of xylem elements in roots, stems and leaf veins [10,33,34], thereby reducing water supply to foliage and amplifying water deficit effects on stomatal conductance and photosynthetic performance. Tissue dehydration also impacts aquaporin (AQP) expression controlling hydraulic conductance of the leaf symplastic compartment [35]. Furthermore, as the concentration of ABA increases in the xylem, AQP activity in the bundle sheath cells is down-regulated, thereby reducing water flow into the leaf as demonstrated by Shatil-Cohen et al. [21].

We analysed the impact of water deficit on plant water status, gas exchange and hydraulic conductance on saplings of silver birch (*Betula pendula* Roth) under artificially manipulated air humidity in field conditions. Silver birch is distributed widely over almost all of Europe, and in northern Europe it is among the most important commercial tree species. Because trees growing in moist atmosphere experience less water loss and have higher stomatal openness, we hypothesize that physiological characteristics in trees acclimated to higher humidity exhibit higher susceptibility to rapidly-induced water deficit. The primary aim of this study was to test this hypothesis experimentally. Secondly we tested whether the putative trade-off between plant hydraulic capacity and water-use efficiency (WUE) is observable on a short time scale. We aimed this study to broaden the understanding of the ability of trees to acclimate with the increasing atmospheric humidity predicted for northern Europe.

## Results

### Effects of air humidification and rapidly-imposed water deficit

**Figure 1:**
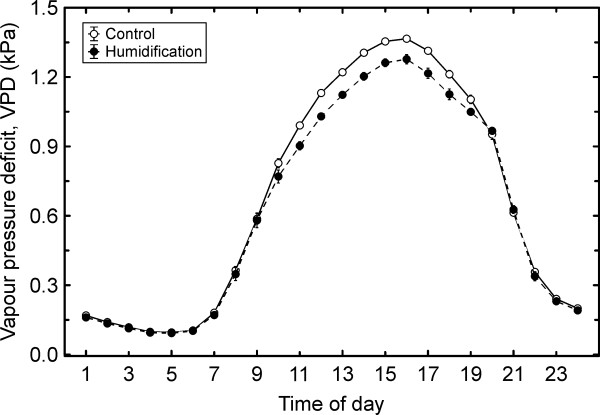
**Daily variation of mean atmospheric water vapour pressure deficit (VPD) in June and July 2010.** The error bars denote S.E.

**Figure 2:**
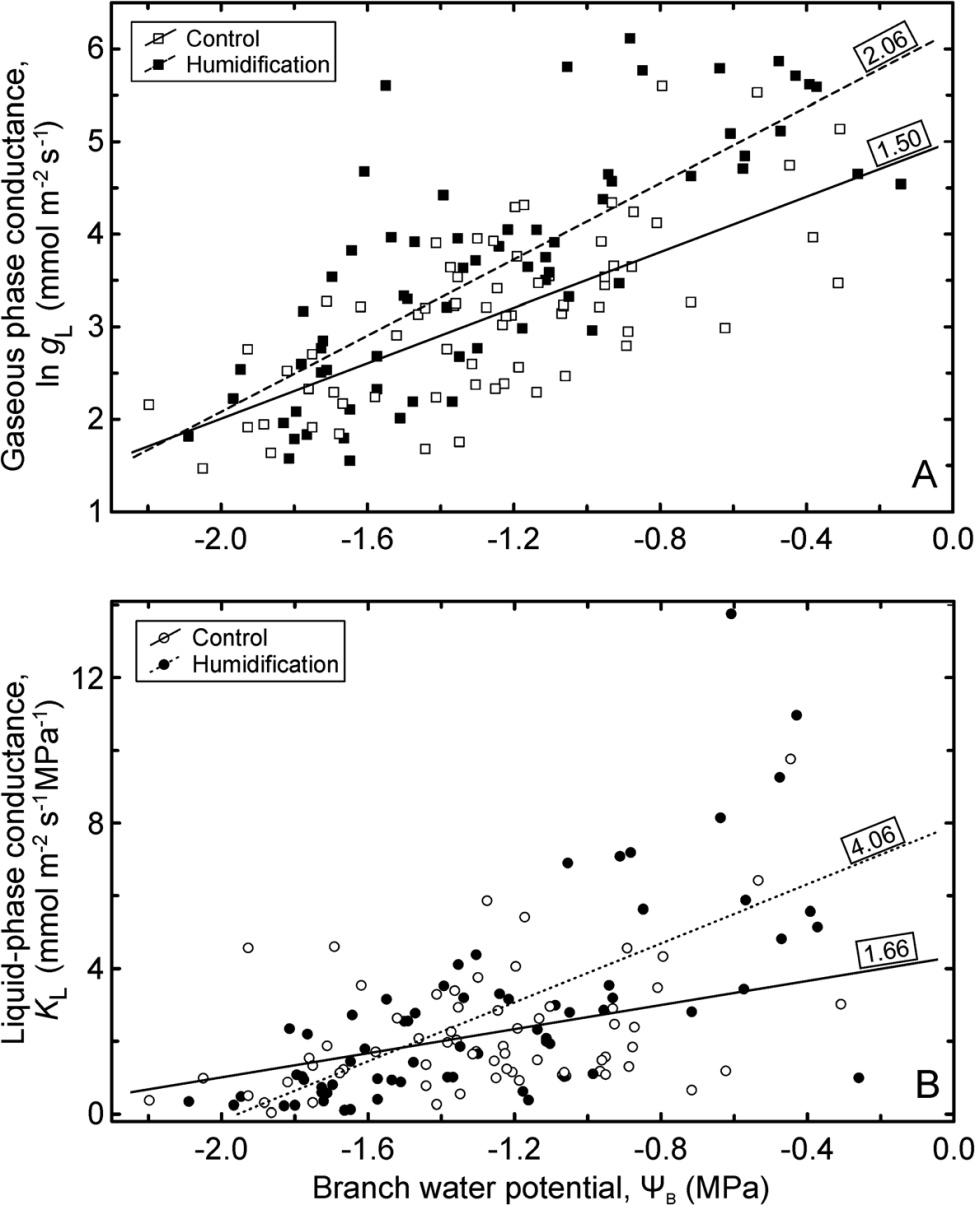
**Branch water potential (Ψ**_**B**_**) versus leaf conductance to water vapour (*g***_**L**_**; A) and leaf hydraulic conductance (*K***_**L**_**; B) in control and humidified trees.** The numbers by the regression lines indicate the respective slopes.

The air humidification caused a decrease of up to 10% in atmospheric water vapour pressure deficit (VPD) during the misting application (Figure 1). ANCOVA revealed that the humidification treatment influenced (*P* < 0.05) most of the studied characteristics (Table 1). The strongest effects were observed for leaf conductance to water vapour (*g*_L_) and leaf water potential (Ψ_L_), whereas leaf temperature (*T*_L_), ratio of intercellular to ambient CO_2_ concentrations (*C*_i_/*C*_a_), net photosynthetic rate (*A*_n_) and intrinsic water-use efficiency (IWUE) remained unaffected by the manipulation. The rapidly-induced water deficit, quantified by leaf (Ψ_L_) or branch water potential (Ψ_B_), had a highly significant (*P* < 0.001) effect on all studied parameters. Except for leaf hydraulic conductance, *K*_L_, inclusion of Ψ_B_ into the analysis model resulted in a considerably better fit than inclusion of Ψ_L_. An analysis of sensitivity of the physiological parameters to changes in plant water status (d*x*/dΨ_B_), estimated by slopes of the corresponding linear regressions, revealed that almost all variables of trees grown under elevated atmospheric humidity (**H** treatment) tended to respond more sensitively to water deficit. However, in only two cases the corresponding slopes differed significantly between the treatments (Figure 2): *g*_L_ (*P* < 0.05) and *K*_L_ (*P* < 0.01). In order to compare the *g*_L_ and *K*_L_ responses to each other, we normalised the absolute values with corresponding means and analysed sensitivity of the normalised *g*_L_ and *K*_L_ (values of both characteristics below or above 1) to developing water deficit. *K*_L_ declined 2.3 times (*P* < 0.01) and *g*_L_ 1.4 times (*P* < 0.05) faster in humidity-treated trees compared to the control with decreasing Ψ_B_.

**Table 1:** Results of ANCOVA for effects of the humidification treatment and fast-imposed water deficit on leaf water status, temperature, gas exchange and hydraulic conductance (N = 117–124)

**Dependent variable**	**Effect**	**Statistical significance**	**Partial *η***^**2**^
Leaf water potential, Ψ_L_	Treatment	*P* < 0.001	0.090
	Branch water status	*P* < 0.001	0.763
Leaf temperature, *T*_L_	Treatment	*ns*	-
	Branch water status	*P* < 0.001	0.246
Leaf conductance to water vapour, *g*_L_	Treatment	*P* < 0.001	0.101
	Branch water status	*P* < 0.001	0.544
Transpiration rate, *E*	Treatment	*P* < 0.001	0.088
	Branch water status	*P* < 0.001	0.401
	Leaf temperature	*P* < 0.001	0.127
Stomatal conductance, *g*_S_	Treatment	*P* = 0.026	0.041
	Branch water status	*P* < 0.001	0.543
	Leaf temperature	*P* = 0.021	0.044
Ratio of intercellular to ambient CO_2_ concentrations, *C*_i_/*C*_a_	Treatment	*ns*	-
	Branch water status	*P* < 0.001	0.338
Net photosynthesis, *A*_n_	Treatment	*ns*	-
	Branch water status	*P* < 0.001	0.518
	Leaf temperature	*P* = 0.039	0.037
Intrinsic water-use efficiency, IWUE	Treatment	*ns*	-
	Branch water status	*P* < 0.001	0.140
Leaf hydraulic conductance, *K*_L_	Treatment	*P* = 0.022	0.039
	Leaf water status	*P* < 0.001	0.433
	Leaf temperature	*P* = 0.004	0.062

*ns*, not significant.

Mean values of the gas exchange and hydraulic characteristics for control (**C** treatment) and humidified trees are presented in Table 2. *E* and *g*_L_ exhibited greater (*P* < 0.05) values in **H** treatment both before branch cutting in the morning and under moderate water deficit (Ψ_L_≥-1.55 MPa). Under moderate water deficit, *T*_L_ was less and *K*_L_ greater in **H** than in **C** branches (*P* < 0.05). The means of other gas exchange parameters showed no difference among treatments. Water deficit developed rapidly after branch cutting, thereby leading to a decline in most parameters, including *K*_L_.

**Table 2:** **Comparison of mean values of physiological characteristics in control (C) and humidified trees (H) before branch cutting (on intact trees) and depending on severity of water deficit (Ψ**_**L**_**<-1.55 MPa versus Ψ**_**L**_**≥-1.55 MPa)**

**Characteristic**	**Before cutting**		**Ψ**_**L**_**≥-1.55 MPa**		**Ψ**_**L**_**<-1.55 MPa**	
	**C**	**H**	**C**	**H**	**C**	**H**
Ψ_L_ (MPa)	-1.07	-1.03	-1.26	-1.25	-1.91	-2.10
Ψ_B_ (MPa)	-0.81	-0.65	-0.98	-0.90	-1.62	-1.56
*T*_L_ (ºC)	26.2	25.4	27.6^*^	26.2^*^	28.8	29.3
*g*_L_ (mol m^-2^ s^-1^)	0.071^*^	0.166^*^	0.044^**^	0.101^**^	0.026	0.060
*E* (mmol m^-2^ s^-1^)	0.92^*^	2.07^*^	0.65^**^	1.33^**^	0.47	1.00
*g*_S_ (mol m^-2^ s^-1^)	0.145	0.237	0.086	0.138	0.046	0.060
*C*_i_/*C*_a_ (dimensionless)	0.70	0.68	0.74	0.71	0.87	0.89
*A*_n_ (μmol m^-2^ s^-1^)	6.47	9.29	4.21	5.96	2.00	2.66
IWUE (μmol mol^-1^)	51.3	48.2	49.3	49.9	35.3	32.8
*K*_L_, (mmol m^-2^ s^-1^ MPa^-1^)	3.65	5.88	2.47^*^	3.78^*^	1.85	1.88
*R*_L_ (dimensionless)	0.32	0.44	-	-	-	-
*K*_S-B_, (mmol m^-2^ s^-1^ MPa^-1^)	2.28^*^	5.26^*^	-	-	-	-
*K*_T_, (mmol m^-2^ s^-1^ MPa^-1^)	1.15^*^	2.36^*^	-	-	-	-

Ψ_L_, leaf water potential; Ψ_B_, branch water potential; *T*_L_, leaf temperature; *g*_L_, leaf conductance to water vapour; *E*, transpiration rate; *g*_S_, stomatal conductance to water vapour; *C*_i_/*C*_a_, ratio of intercellular to ambient CO_2_ concentrations; *A*_n_, net photosynthesis; IWUE, intrinsic water-use efficiency; *K*_L_, leaf hydraulic conductance; *R*_L_, relative leaf hydraulic resistance; *K*_S-B_, soil-to-branch hydraulic conductance; *K*_T_, whole-tree hydraulic conductance. Statistical significance of the difference: ^*^P < 0.05, ^**^P < 0.01.

**Figure 3:**
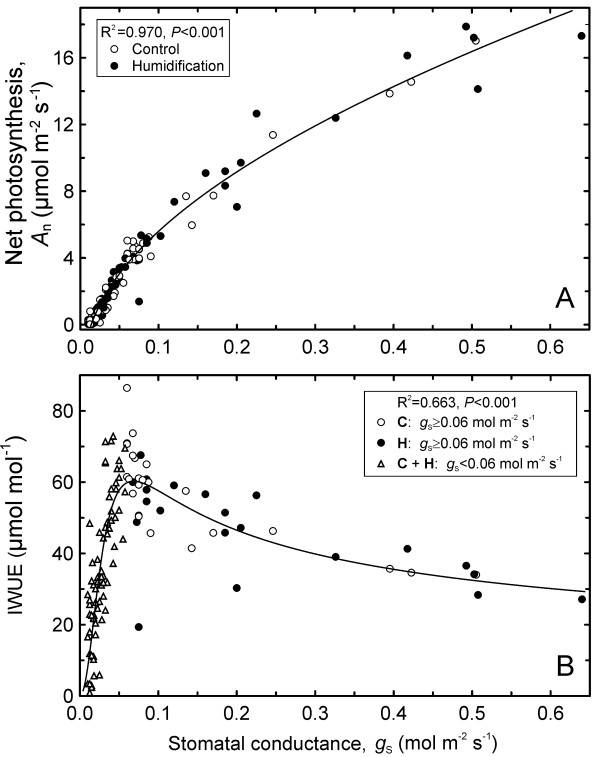
**Stomatal conductance (*g***_**S**_**) versus net photosynthetic rate (*A***_**n**_**; A) and intrinsic water-use efficiency (IWUE; B) across control (C) and humidified trees (H).**

Net photosynthetic rates were strongly correlated with stomatal conductance (*g*_S_; R^2^ = 0.970, *P* < 0.001) across a wide range of stomatal openness for both treatments combined (Figure 3A). At first IWUE increased in response to the rapidly-induced water deficit and attained a maximum of >70 μmol mol^-1^, corresponding to *g*_S_ ~0.06 mol m^-2^ s^-1^ (Figure 3B). When *g*_S_ fell below this value (at Ψ_B_ < -1.0 MPa), IWUE declined very steeply as *A*_n_ decreased more rapidly than *g*_S_. Two characteristics - *T*_L_ and *C*_i_/*C*_a_ - demonstrated opposite trends with increasing water deficit. None of the characteristics differed significantly among the treatments under severe water deficit (Ψ_L_<-1.55 MPa; Table 2).

### Long-term effects of water deficit

**Figure 4:**
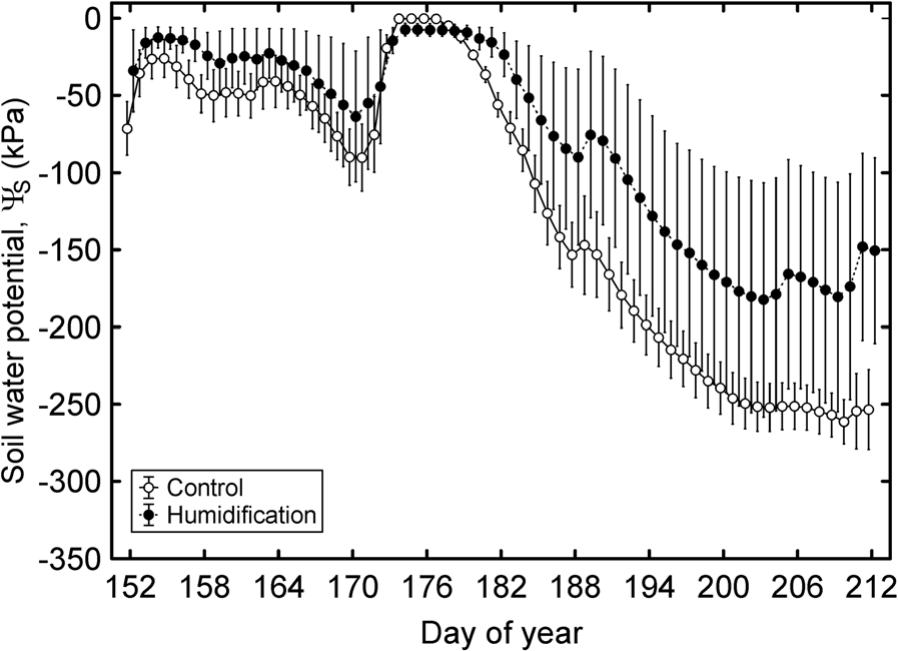
**Mean bulk soil water potential (Ψ**_**S**_**) in control and humidified plots in June and July 2010.** The error bars denote S.E.

Long-term water deficit was imposed by reducing soil water availability due to a moderate drought that developed in July (Table 3; Figure 4). Although the misting application decreased transpirational water loss, bulk soil water potential (Ψ_S_) in **H** plots also underwent substantial decline (dropped to -180 kPa) in July. Inclusion of Ψ_S_ as an index of soil water availability into the analysis models changed the outcome radically: the effect of the humidification treatment became – with one exception – insignificant for all gas exchange and water relations characteristics (Table 4). Only *g*_L_ depended simultaneously on the treatment (*P* = 0.036), rapidly-induced water deficit (Ψ_B_; *P* < 0.001) as well as soil water availability (Ψ_S_; *P* < 0.001). Consequently, the effects of humidification manipulation were to a great extent realized through changes in soil water status. Four characteristics [*g*_L_, *E*, soil-to-branch hydraulic conductance (*K*_S-B_) and whole-tree hydraulic conductance (*K*_T_)] were 2.1–2.3 times greater in humidified trees than in control trees.

**Table 3:** Sums of precipitation (mm) at the FAHM site in June and July

**Month**	**Year**		
	**2008**	**2009**	**2010**
June	79	152	110
July	64	90	33

**Table 4:** Results of ANCOVA for effects of the humidification treatment and fast and long-term water deficit on leaf water status, temperature, gas exchange and hydraulic conductance (N = 117–124)

**Dependent variable**	**Effect**	**Statistical significance**	**Partial *η***^**2**^
Leaf water potential, Ψ_L_	Treatment	*ns*	-
	Branch water status	*P* < 0.001	0.808
	Soil water availability	*P* < 0.001	0.209
Leaf temperature, *T*_L_	Treatment	*ns*	-
	Branch water status	*P* < 0.001	0.246
	Soil water availability	*ns*	-
Leaf conductance to water vapour, *g*_L_	Treatment	*P* = 0.036	0.033
	Branch water status	*P* < 0.001	0.572
	Soil water availability	*P* < 0.001	0.164
Transpiration rate, *E*	Treatment	*ns*	-
	Branch water status	*P* < 0.001	0.413
	Soil water availability	*P* < 0.001	0.145
	Leaf temperature	*P* < 0.001	0.129
Stomatal conductance, *g*_S_	Treatment	*ns*	-
	Branch water status	*P* < 0.001	0.560
	Soil water availability	*P* < 0.001	0.184
	Leaf temperature	*P* = 0.001	0.087
Ratio of intercellular to ambient CO_2_ concentrations, *C*_i_/*C*_a_	Treatment	*ns*	-
	Branch water status	*P* < 0.001	0.338
	Soil water availability	*ns*	-
Net photosynthesis, *A*_n_	Treatment	*ns*	-
	Branch water status	*P* < 0.001	0.526
	Soil water availability	*P* < 0.001	0.122
	Leaf temperature	*P* = 0.006	0.067
Intrinsic water-use efficiency, IWUE	Treatment	*ns*	-
	Branch water status	*P* < 0.001	0.140
	Soil water availability	*ns*	-
Leaf hydraulic conductance, *K*_L_	Treatment	*ns*	-
	Leaf water status	*P* < 0.001	0.465
	Soil water availability	*P* = 0.003	0.064
	Leaf temperature	*P* = 0.002	0.073

*ns*, not significant.

In fact, the differences in physiological characteristics between the treatments recorded on intact branches in the morning (Table 2) reflect co-effects of the air humidification and long-term soil water deficit. The responses of *g*_L_ and *K*_L_ to variation in Ψ_B_ were analysed also separately for the data obtained before and after cutting branches, and for moister (Ψ_S_>-218 kPa) and drier soil conditions (Ψ_S_≤-218 kPa). Before cutting, neither of the response slopes differed between the treatments; after cutting, both slopes differed significantly between the treatments (*g*_L_, *P* < 0.05; *K*_L_, *P* < 0.01). d*g*_L_/dΨ_B_ and d*K*_L_/dΨ_B_ showed no difference within treatments between the different soil moisture ranges.

### Liquid versus gaseous phase conductance

**Figure 5:**
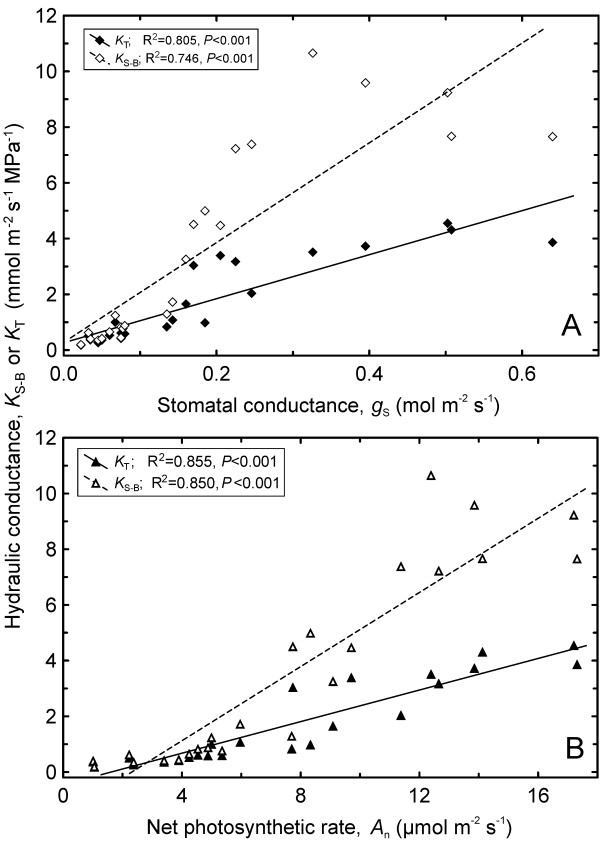
**Co-ordination between gaseous and liquid-phase conductances.** Stomatal conductance to water vapour (*g*_S_; **A**) and net photosynthetic rate (*A*_n_; **B**) versus soil-to-branch hydraulic conductance (*K*_S-B_) and whole-tree conductance (*K*_T_) across humidification and control treatments.

Changes in *K*_L_ were co-ordinated with those in both stomatal conductance and net photosynthesis, while the relationships were substantially stronger for humidified trees. Specifically, R^2^ in **C** treatment was 0.264 and 0.293 for *g*_S_ and *A*_n_, respectively. In **H** treatment the respective R^2^ values were 0.583 and 0.601 (for all cases *P* < 0.001). *g*_S_ and *A*_n_ were associated considerably more strongly with *K*_S-B_ (R^2^ = 0.75-0.85) and *K*_T_ (R^2^ = 0.80-0.85; Figure 5). IWUE in intact branches declined with increasing hydraulic capacity: with *K*_L_ (R^2^ = 0.204, *P* < 0.05), *K*_S-B_ (R^2^ = 0.356, *P* < 0.01) as well as *K*_T_ (R^2^ = 0.356, *P* < 0.01). There was no statistical relationship between *K*_L_ and IWUE across the whole data sets (i.e., throughout the whole range of water deficit). The reliability of gasometric measurements was proved by an excellent accord among the readings obtained with different instruments: although *g*_S_ and total leaf conductance (*g*_L_) were measured on different leaves and under different conditions (controlled versus ambient conditions, respectively), the two characteristics exhibited a near perfect concordance (R^2^ = 0.944 for **C** trees, R^2^ = 0.901 for **H** trees, for both *P* < 0.001).

## Discussion

### General responses to water deficit

The rapidly-induced water deficit had highly significant (*P* < 0.001) effect on all parameters measured at the leaf level (Table 1). Under moderate water deficit (Ψ_L_≥-1.55 MPa) leaf conductance to water vapour, transpiration rate and leaf hydraulic conductance were significantly (*P* < 0.05) higher in trees grown at elevated air humidity than in control trees. These differences are attributable to higher initial values (a result of long-term effects) and probably also to larger branch internal water storage in **H** treatment under moderate drought, although statistically not proven by the Ψ_B_ data. Leaf temperature, on the contrary, was higher (*P* < 0.05) in **C** trees due to the diminished transpiration. Under severe water deficit (Ψ_L_<-1.55 MPa) the treatments showed no difference in any of the characteristics (Table 2).

Two characteristics - *g*_L_ and *K*_L_ - exhibited a significantly steeper decline with increasing water deficit in **H** treatment than in the control, indicating higher susceptibility to weather fluctuations of trees grown under increased RH. The observed stomatal responses are primarily associated with impact of rapidly-induced water deficit and obviously driven by hydraulic signals, because d*g*_L_/dΨ_B_ did not differ between the treatments in intact branches and did not depend on soil water status if the data was analysed separately in subsets. Thus, the effect of soil drying is secondary. Various mechanisms are suggested as signalling cues to initiate or enhance ABA biosynthesis, including hydraulic signals [36]. The priority of hydraulic versus metabolic stimuli is considered fundamentally important in preventing plant desiccation and is maintained in stomatal control through vascular plant phylogeny [37,38]. However, the apparent change in stomatal sensitivity to branch water status induced by the humidity manipulation could be due to differences in the leaf-borne ABA levels, as previous reports describe that endogenous ABA concentrations in leaves grown for a long time under high humidity are lower than under moderate humidity [11,12]. Also fast de novo synthesis or conversion of inactive conjugates of ABA [15,16] in shoot vascular tissues triggered by branch dehydration cannot be dismissed. Although studies on *Arabidopsis thaliana* provide crucial information on stomatal responses, species-specific differences exist, especially when the plants are exposed to simultaneously changing environmental factors [39].

Thus, our experiment supports the first hypothesis that trees acclimated to higher humidity exhibit greater sensitivity to rapidly-induced water deficit with respect to two functional traits. However, these changes have different consequences on plant water status. The reduction of *g*_L_ helps to limit water loss, slows down further Ψ_L_ falling and prevents runaway xylem embolism. The impact of decreasing *K*_L_ is opposite – leaf water supply declines causing Ψ_L_ to fall. Birch trees showed differential changes in these two fundamental traits due to the experimental manipulation: the humidity-treated trees exhibited substantially faster water deficit-driven reduction in *K*_L_ than in *g*_L_ if compared to the control. Thus, greater risk of leaf dehydration and xylem dysfunction is probably imposed on the trees grown under higher atmospheric humidity in case of sudden weather extremes, because strict stomatal control over water loss is a crucial factor in preventing water deficit-induced xylem cavitation [13]. Plant hydraulic conductance does not limit stomatal openness under moist weather conditions, but it could become crucial in climate extremes (severe drought, disastrous heat wave), which are scarcely predictable and yet will become more frequent in the future [8]. Among ecosystems, forests are particularly sensitive to climate change, because the long life-span and conservative structure of the water-conducting system of trees do not allow rapid acclimation to environmental fluctuations [3].

The air humidification manipulation affected most of the studied characteristics, but not IWUE (Table 1), unlike the soil humidity manipulation reported by Possen et al. [40]. In some species even long-term soil drought does not affect IWUE if *A*_n_ and *g*_S_ decrease with equal rates [41]. The inclusion of Ψ_S_ in the analysis models excluded the treatment effect (Table 4), suggesting that the impact of experimental manipulation in droughty summer (Table 3) is realized largely through changes in soil water status (i.e. due to higher Ψ_S_ in **H** treatment). Only leaf conductance to water vapour (*g*_L_) depended simultaneously on the treatment (*P* = 0.036), rapidly-induced water deficit (*P* < 0.001) and soil water availability (*P* < 0.001). However, *g*_L_ in **H** trees demonstrated higher sensitivity to water deficit, i.e. an opposite trend to that observed by Fanourakis et al. [4]. Weak stomatal control could be a consequence of the low transpiration in plants grown continuously under high RH (>85%). The degree of stomatal acclimation depends on both the duration and timing of exposure to high RH during leaf development, while determinative is just a stage of leaf expansion completion [4]. In silver birch, elevated atmospheric humidity had the widest consequences on stomatal regulation, as the effects extended beyond that of soil water availability. This is an important point in view of climate change: Roelfsema and Hedrich [20] argue that stomata will play an essential role in the adaptation of plants to climate change, because of their interrelated roles in CO_2_ uptake and release of water. As for *g*_S_, we observed less pronounced response (compare Tables 1 and 4), obviously because of its being measured under artificial conditions (constant irradiance, temperature and air humidity).

### Changes in plant hydraulic traits

The air humidity manipulation led to higher soil water availability (Figure 4) in **H** treatment due to reduced transpirational water loss [5,7] under low VPD during the misting application (Figure 1). This resulted in higher hydraulic capacity of the trees grown in more humid environment, i.e. a long-term effect (Table 2). This response was observed under the moderate drought in July 2010 (Table 3). By contrast, we did not observe unequivocal shifts in hydraulic traits in the rainy summer of 2009: *K*_L_ decreased, while hydraulic conductance of root systems (*K*_R_) and leaf-specific conductivity of stem-wood increased in response to elevated RH [42]. The present study revealed some alleviating effect of elevated RH under moderate drought, and the plant response to increased air humidity seems to differ depending on prevailing weather conditions. Nor can we dismiss increased xylem vulnerability and possible hydraulic dysfunction under unexpected severe drought, although on average the climate will become more humid at high latitudes [2,3].

The differences in *K*_S-B_ and *K*_T_ observed on intact trees in 2010 likely ensued from xylem cavitation in response to differential soil drying (i.e. a long-term effect) in the treatments. The differences in *K*_L_ resulted from rapidly-imposed water deficit rather than soil water availability, because *K*_L_ measured on intact branches showed no significant difference between the treatments (Table 2) and d*K*_L_/dΨ_B_ was invariant of soil water status. *K*_S-B_ demonstrated a greater intertreatment variation compared to *K*_L_ - by a factor of 2.3 versus 1.6, respectively. This is attributable to greater susceptibility of root xylem than of shoot xylem to water stress-induced embolism [33,43]. Domec et al. [44] reported that *K*_R_ declines faster than *K*_L_ as soil dries. The increasing resistance between soil and trunk has been shown to be the main cause of *K*_T_ decline and has also the highest weight in the stomatal control [45]. We cannot exclude also concurrent mechanisms responsible for the differences in the decline of *K*_S-B_ versus *K*_L_, such as that associated with contribution of apoplastic versus cell-to-cell route to liquid water transport under water deficit. When transpiration stream is attenuated, plasma membrane AQPs are upregulated, the membrane water permeability increases and transcellular water flux becomes much more significant [46]. One must consider that the soil-to-branch pathway represents predominantly an apoplastic route, while in leaves the contributions of the two routes to the total hydraulic resistance are of the same magnitude [47]. Nevertheless, Johnson et al. [48,49] measured *K*_L_ concurrently with ultrasonic acoustic emissions in dehydrating leaves of several woody species and presented reliable evidence that xylem embolism is a primary factor in dehydration-induced declines in leaf hydraulic conductance. Findings of Nardini et al. [50] highlight the role of regulation of *K*_L_ in plant acclimation suggesting that leaf resistance to drought-induced hydraulic dysfunction is a key to plant survival and competition even over limited geographical ranges.

### Co-ordination between gas exchange and hydraulic traits

Net photosynthetic rate (*A*_n_) and stomatal conductance (*g*_S_) in silver birch were positively correlated with plant hydraulic characteristics (Figure 5), whereas gas exchange parameters were considerably more strongly associated with *K*_S-B_ or *K*_T_ than with *K*_L_. This result confirms that maximum *g*_S_ and *A*_n_ depend on hydraulic conductance of the whole soil-to-leaf pathway (expresses potential capability for leaf water supply) rather than solely on that of the leaf [45,51,52].

The rapidly-imposed water deficit affected (*P* < 0.001) all parameters measured at the leaf level, showing substantially stronger association with Ψ_B_ than with Ψ_L_ (Table 1). Thus, the gas exchange and stomatal conductance of silver birch are determined by direct water availability to the leaf, estimated by Ψ_B_ in the petiole insertion point, rather than by the current leaf water status (Ψ_L_) itself. The relationship between gas exchange and Ψ_B_ is probably mediated by stem hydraulic capacitance, because the internal water storage in trees plays a role in mitigating diurnal fluctuations in plant water status caused by transpirational water losses [14,53]. So, plants with a great capacity to avoid high stem water deficits during periods of high transpiration tend to have a relatively risky stomatal strategy and maintain higher midday *g*_S_[17]. On the other hand, our results support the idea that stomatal openness is regulated in a way to prevent primarily dysfunction of stem xylem, as proposed by Meinzer et al. [14]. This is likely a general trait for broad-leaved trees, as recently reported for a number of subtropical tree species [17].

Leaf gaseous phase conductance began to decrease simultaneously with *K*_L_ in response to the rapidly-imposed water deficit, i.e. with no threshold level in the water potential range experienced in the present study. This result coincides with that obtained on leaves of *Quercus*, *Pinus* and *Pseudotsuga* species [54]. Although the field measurements under uncontrolled conditions did not allow construction of vulnerability curves, our data imply narrow hydraulic safety margin existing in silver birch (the 50% decline of *K*_L_ was observed at about -1.2 MPa), a characteristic of angiosperm species [55]. Blackman et al. [56] sampled 20 phylogenetically disparate woody angiosperms and found that the greater the water potential inducing a 50% loss in *K*_L_, the narrower the safety margin. This trait suits well with general life strategy of a fast-growing pioneer species, such as *B. pendula*. In this context the present result is consistent with our previous findings: stomatal sensitivity of sun leaves of *B. pendula* to atmospheric VPD (80 mmol m^-2^ s^-1^ ln(kPa)^-1^[57]) exceeds the corresponding mean of angiosperms (73 mmol m^-2^ s^-1^ ln(kPa)^-1^[55]). Contrary to the paradigm that isohydric species avoid cavitation, it has been revealed that relatively isohydric species tend to experience far greater cavitation and refilling of xylem on a daily basis than anisohydric species, the benefit of which is enhanced capacitance for use in transpiration [58].

Silver birch has been reported to be able for efficient acclimation to lack of water, including adjustment of WUE [40]. Thus, the drought developed in Estonia in summer 2010 was not severe enough to induce significant changes in photosynthetic water-use efficiency (IWUE; Table 4). Our earlier studies [57,59] performed on large birch trees growing in a natural forest stand revealed the opposing height-related trends in IWUE and soil-to-leaf hydraulic conductance (*K*_T_) within tree crowns at sufficient light intensities, suggesting a trade-off between water transport and use efficiencies. The inverse relationships between hydraulic characteristics and IWUE found in this study suggest that the respective trade-off between hydraulic capacity and WUE occurs in silver birch both at the leaf (*K*_L_) and whole-plant levels (*K*_T_). The trade-off reflects co-ordinated adjustment of plant gas exchange and hydraulic system to long-term water deficit, but not a response to rapidly-imposed interference; therefore, the converse relation was discovered only in intact branches. Hence, it is always necessary to consider time scales when analysing trends in plant WUE. Abril and Hanano [19] indicated that WUE in Mediterranean woody species reduces during the day by water stress, but it increases as seasonal drought proceeds.

## Conclusions

Our results support the hypothesis that physiological traits in trees acclimated to higher air humidity exhibit higher sensitivity to rapid water deficit with respect to two characteristics - leaf conductance to water vapour and leaf hydraulic conductance. Disproportionate changes in sensitivity of stomatal versus leaf hydraulic conductance to water deficit might impose greater risk of desiccation-induced hydraulic dysfunction on the plants, grown under high RH, in case of sudden weather fluctuations. We failed to discover a short-term trade-off between plant hydraulic capacity and photosynthetic water-use efficiency. The impact of air humidity manipulation was realized principally through changes in soil water availability, while the treatment may have different effects on plant functioning depending on weather conditions prevailing during the growing season.

## Methods

### Study area and environmental variables

The studies were carried out on 5-year-old silver birch (*B. pendula*) trees in an experimental forest plantation at the Free Air Humidity Manipulation (FAHM) site, situated in Rõka village (58°14′N, 27°17′E, 40–48 m ASL), Eastern Estonia, representing a hemiboreal vegetation zone. The long-term average annual precipitation in the region is 650 mm and the average temperature is 17.0°C in July and -6.7°C in January. The growing season lasts 175–180 days from mid-April to October. The soil is a fertile Endogenic Mollic Planosol (WRB) with an A-horizon thickness of 27 cm. Total nitrogen content is 0.11-0.14%, C/N ratio is 11.4, and pH is 5.7–6.3.

Three sample plots served as control areas (**C** treatment) and three plots were humidified (**H** treatment) using the computer-operated FAHM system. The system integrates two different technologies - a misting technique to atomize/vaporise water and a FACE-like technology to mix humidified air inside the plots, enabling relative humidity of the air (RH) to be increased by up to 18% over the ambient level during humidification treatment, depending on the wind speed inside the experimental stand. The humidification was applied in daytime 6 days a week throughout the growing period if ambient RH was <75% and mean wind speed <4 m s^-1^. As a long-term average, RH was increased by 7–8%. A detailed description of the FAHM site and technical setup has been presented by Kupper et al. [5]. The manipulation was started in June of 2008; gas exchange and hydraulic measurements were performed on 15 **H** and 15 **C** trees in June and July of 2010. Environmental variables measured continuously were air temperature (*T*_A_) and relative humidity (RH) with HMP45A humidity and temperature probes (Vaisala, Helsinki, Finland), precipitation with TR-4 tipping bucket rain gauges (Texas Electronics, Dallas, TX), bulk soil water potential (Ψ_S_) with EQ2 equitensiometers (Delta-T Devices, Burwell, UK) at depths of 15 and 30 cm. The readings of the sensors were stored as 10 minute average values with a DL2e data logger (Delta-T Devices).

### Gasometric and hydraulic measurements

One sample branch (mean height above the ground 140±9.3 cm for **C** trees and 138±8.4 cm for **H** trees) per tree from the middle third of the crown was selected for gasometric and hydraulic measurements. Two branches, one from **C** and another form **H** treatment, were sampled simultaneously using two instruments. Net photosynthetic rate (*A*_n_), stomatal conductance to water vapour (*g*_S_) and ratio of intercellular to ambient CO_2_ concentrations (*C*_i_/*C*_a_) were measured with a LCpro+ portable photosynthesis system (ADC BioScientific, Hoddesdon, UK) on four or five leaves per branch at a saturating photosynthetic photon flux density (1196 μmol m^-2^ s^-1^) applying constant CO_2_ concentration (*C*_a_ = 360 μmol mol^-1^), air humidity (water vapour pressure 15 mbar) and temperature (25ºC). Leaf conductance to water vapour (i.e. total gaseous phase conductance, *g*_L_), transpiration rate (*E*) and leaf temperature (*T*_L_) were measured on six leaves per branch with a LI-1600M steady-state diffusion porometer (Li-Cor, Lincoln, NE) at ambient conditions. Intrinsic water-use efficiency (IWUE) was calculated as the ratio of *A*_n_ to *g*_S_[41,60]. Bulk leaf water potential (Ψ_L_) was determined in four detached leaves by the balancing pressure technique using a Scholander-type pressure chamber simultaneously with gas exchange measurements. Xylem water potential of the branches (Ψ_B_) was estimated by applying the bagged leaves technique, sampling two leaves per branch at each measurement time, prepared the previous evening. Water potential of the non-transpiring (bagged) leaves, presumed to have equilibrated with the xylem water potential of the branch proximal to the petiole, was taken as an estimate of Ψ_B_. The first measurement series was performed on intact branches in the morning immediately before branch cutting. Then the sample branches were cut off and allowed to dehydrate in open-air conditions in order to generate a rapidly-imposed water deficit. The next four measurement series were conducted within ~3 h after cutting. All measurements were done on dry leaves under non-misting conditions: on intact branches in the morning before misting started and after that outside the experimental plots.

Hydraulic conductance of leaves (*K*_L_) was estimated by the evaporative flux method under steady-state conditions and was calculated according to the Ohm’s law analogy:

(1) $$K_{L}=\frac{E}{\Psi_{B}-\Psi_{L}}\;,$$

where *E* is the evaporative flux. As *E* is expressed per unit leaf area, values of *K*_L_ have been scaled by leaf area. *K*_L_ was standardized for the dynamic viscosity of water at 28ºC. Soil-to-branch (*K*_S-B_) and whole-tree hydraulic conductance (*K*_T_) were calculated analogically based on water potential drops across the corresponding segments (Ψ_S_-Ψ_B_ and Ψ_S_-Ψ_L_, respectively). *K*_S-B_ and *K*_T_ were left unstandardized, because of variable temperature along these long transport pathways.

### Data analysis

Statistical data analysis was carried out using Statistica, Vers. 7.1 (StatSoft Inc., Tulsa, OK). Effects of air humidification (treatment), rapidly-imposed (estimated by Ψ_L_ or Ψ_B_) and long-term water deficits (estimated by Ψ_S_) on leaf gas exchange and hydraulic conductance were analysed by applying analysis of covariance (ANCOVA). We acknowledge that data from such field experiments do not allow strict separation of the rapid and long-term effects of water deficit, however, this approach was encouraged by absence of differences both in Ψ_L_ and Ψ_B_ between the treatments before branch cutting in the morning (see Table 2). ‘Treatment’ was treated as a categorical predictor, while Ψ_S_, *T*_L_ and Ψ_L_ or Ψ_B_ were included in the analysis model as covariates; type IV sums of squares were used in the analysis. The ANCOVA was performed in two stages: first, analysis of the treatment and rapidly-imposed water deficit effects; second, addition of the effect of the long-term water deficit. Statistically insignificant covariates were removed from the final models. Effect sizes were assessed by partial eta-squared ($\eta_{\text{partial}}^{2}$) defined as the ratio of variance accounted for by an effect and that effect plus its associated error variance [61]:

(2) $$\eta_{\text{partial}}^{2}=\frac{{\mathrm{SS}}_{\text{effect}}}{SS_{\text{effect}}+SS_{\text{error}}}\;,$$

where SS_effect_ is the sum of squares for given effect and SS_error_ is the sum of squares for the respective error term.

## Abbreviations

Treatments: C: Control trees grown in natural air humidity; H: Trees grown in elevated air humidity; An: Net photosynthetic rate; ABA: Abscisic acid; AQP: Aquaporin; Ci/Ca: Ratio of intercellular to ambient CO_2_ concentrations; E: Transpiration rate; gL: Leaf conductance to water vapour; gS: Stomatal conductance; IWUE: Intrinsic water-use efficiency; KL: Leaf hydraulic conductance; KR: Root hydraulic conductance; KS-B: Soil-to-branch hydraulic conductance; KT: Soil-to-leaf or whole-tree hydraulic conductance; RL: Relative leaf hydraulic resistance; RH: Relative humidity of air; TA: Air temperature; TL: Leaf temperature; VPD: Atmospheric water vapour pressure deficit; WUE: Water-use efficiency; ΨB: Branch water potential; ΨL: Leaf water potential; ΨS: Bulk soil water potential.

## Competing interests

The authors declare that they have no competing interests.

## Authors’ contributions

AS designed and performed the experiment, and wrote the manuscript. AN, EÕP and PK performed the experiment, analyzed the data and revised the paper. All authors read and approved the final manuscript.
